# Plasma Metabolomic Profiling Reveals Preliminary Biomarkers of Pork Quality Based on pH Value

**DOI:** 10.3390/foods11244005

**Published:** 2022-12-11

**Authors:** Linyuan Shen, Jianfeng Ma, Haodi Zhou, Lei Chen, Jie Tang, Kaige Zhang, Ye Zhao, Lili Niu, Shunhua Zhang, Anan Jiang, Jinyong Wang, Zongyi Guo, Xuewei Li, Yiwu Chen, Mailin Gan, Li Zhu

**Affiliations:** 1 Department of Animal Science, College of Animal Science and Technology, Sichuan Agricultural University, Chengdu 611130, China; 2 Farm Animal Genetic Resource Exploration and Innovation Key Laboratory of Sichuan Province, Sichuan Agricultural University, Chengdu 611130, China; 3 Chongqing Academy of Animal Science, Chongqing 402460, China; 4 Key Laboratory of Zoonosis Research, Ministry of Education, College of Animal Sciences, Jilin University, Changchun 130062, China

**Keywords:** pork meat, meat quality, pH, metabolomics, plasma biomarkers

## Abstract

This study aimed to identify biomarkers for pork quality evaluation. Firstly, the correlation between indicators of pork quality evaluation was investigated. The pH of pork meat at 45 min post slaughter showed a significant negative correlation with meat color indicators (*r*: −0.4868–−0.3040). Subsequently, porcine plasma samples were further divided into low pH (pH = 6.16 ± 0.22) or high pH (pH = 6.75 ± 0.08) groups. Plasma metabolites in both sample groups were investigated using untargeted metabolomics. In total, 90 metabolites were recognized as differential metabolites using partial least squares discriminant analysis. Pathway enrichment analysis indicated these differential metabolites were enriched in amino acid metabolism and energy metabolism. Correlation analysis revealed that creatinine, L-carnitine, D-sphingosine, citraconic acid, and other metabolites may constitute novel plasma biomarkers with the pH value of pork meat. The current study provides important insights into plasma biomarkers for predicting pork quality based on pH value.

## 1. Introduction

People’s living conditions are gradually improving and there has been a continuously increasing demand for high-quality and healthy pork [1]. The improvement in meat quality is important not only for the food technologists, but also for the animal producers. Pork meat quality is influenced by a variety of intrinsic and extrinsic factors, including genetic background, nutrition, and feeding schemes [2]. Besides, pre-slaughter stress and post-slaughter handling are also an important factor. Recently, the major gene likely involved in determining pork meat quality has been identified [3,4]. Pork meat quality traits are usually determined after slaughter in order to determine animal breeding value. However, although accurate and reliable, this evaluation method is destructive, time-consuming, and costly. Consequently, loss of elite genetic resources might happen, which constitute a challenge for breeders [5]. In this context, determining biomarkers for predicting pork meat quality in living animals is promising.

Metabolomics is an emerging technique that enables identifying metabolites, commonly low-molecular-weight metabolites (<1 kDa), in tissues, cells, or fluids. Based on the purpose of the metabolomic analysis, it can be further categorized into untargeted and targeted metabolomics [6]. Targeted metabolomics is often used to quantify one or several classes of metabolites, whereas untargeted metabolomics enables the determination of a global metabolite profile [7]. Metabolomics is a powerful and promising tool for identifying phenotypes in livestock, thus providing new approaches to decoding the interplay between genetic and environmental factors [8]. Eight metabolites possibly related to feed efficiency in cattle have been identified using metabolomics [9]. In addition, distinctive metabolite profiles were determined in four commercial chicken breeds using the untargeted metabolomic approach [10], which enabled the identification of differential metabolites among chicken breeds. Metabolomics are good approaches and constrained by cost and difficulty. However, metabolomics can potentially be a tool for exploring biomarkers to predict pork meat quality, especially those that do not require slaughter.

Here, we investigated the correlation among pork meat quality traits of longissimus thoracis et lumborum (LTL) muscle tissue. Moreover, plasma metabolite profiles were explored based on untargeted metabolomics using ultra-high-performance liquid chromatography coupled with mass spectrometry (UHPLC–MS/MS).

## 2. Materials and Methods

### 2.1. Ethics Statement

This work was a routine commercial trade and did not require an ethics statement. The pigs were slaughtered according to national guidelines (GB/T 17236–2008).

### 2.2. Animals and Handling

A total of 202 Yorkshire gilts with a mean weight of 114 ± 16 kg were slaughtered and employed in the present study. These pigs have reached the marketed age. The animals used in this work were reared on a commercial farm. All pigs were non-carriers of the halothane gene and clinically healthy. These pigs were feed under the same comfortable environmental conditions. The diet conditions of pigs met the recommendations of the National Research Council (NRC, 2012). All pigs were slaughtered according to a method previously reported [11]. Animals were not fed for 24 h prior to slaughter.

### 2.3. Sample Collection

Blood samples collected with EDTA were obtained from the anterior vena cava 1 h prior to slaughter. Then, blood samples were centrifuged at 3000× *g* for 10 min at 4 °C, and the supernatant was collected as plasma. The plasma was aliquoted and stored at −80 °C. We stored half of the carcasses in cold storage at 4 °C for measuring pH value and meat color indicators after slaughter. The muscle sample excised from the carcasses was used to measure other indicators. A portion of sample was immediately frozen in liquid nitrogen and stored at −80 °C. A total of 202 LTL muscle samples were further employed in the subsequent meat quality analysis.

### 2.4. Meat Quality Traits

Meat quality traits were determined according to previous studies conducted by our group [1,12], and a summary is provided below.

The pH value of LTL muscle tissue was measured 45 min (pH_1_) and 24 h (pH_2_) postmortem using a portable temperature compensated pH meter (model 720A; Orion Research Inc., Boston, MA, USA). The pH meter was calibrated using standard solutions (pH 4.6 and pH 7.0). Then, pH_1-2_ value was obtained as a result of pH_1_ minus pH_2_.

Meat color measurements were determined at 45 min and 24 h after slaughter using a Minolta CR-300 colorimeter (Minolta Camera, Osaka, Japan) equipped with an 8 mm aperture, D65 illuminant source, and 10° standard observer. Meat color data (L*, lightness; a*, redness; b*, yellowness) were presented as the average of three measurements.

Cooking loss was determined in meat 1 h after removal from the carcass; the meat sample was steamed for 30 min, cooled to room temperature, and weighed; cooking loss was defined as the percentage of weight loss during cooking.

The plastic-bag method was used to measure drip loss of meat samples [13]; 30 g (2 × 3 × 5 cm^3^) of meat samples was minced into cubes in parallel to the direction of muscle fibers and then placed in plastic bags; the percentage of weight loss after 48 h of storage at 4 °C was calculated.

In addition, shear force of samples was measured using a texture analyzer (TA.XT. Plus, Stable Micro Systems, Godalming, UK) equipped with a Warner–Bratzler shearingattachmen; LTL muscle tissue was removed after storage at 4 °C for 72 h and cooked under constant temperature (80 °C) in a water bath. When the internal core temperature of meat samples reached 70 °C, meat samples were cooled to room temperature, then drained and weighed. Three cylindrical cores (10 mm × 20 mm) were cut along muscle fiber direction. Cylindrical meat samples were sheared using the hot dog shearing procedure. The blade speed parameters were set as follows: pre-test, 2 mm/s; test, 2 mm/s; and post-test, 10 mm/s.

Finally, the water-holding capacity (WHC) was determined by the filter paper press method [14]; muscle samples (approximately 2 g) were placed on absorbent Whitman 1 filter paper, covered with another piece of filter paper, then pressed with a 10 Kg weight for 5 min. WHC was defined as sample weight after pressing as a percentage of weight before pressing. All samples were analyzed in triplicates for each indicator. The results were expressed as the mean value of three measurements.

### 2.5. Determination of Metabolite Concentration

To measure metabolite concentration, the following commercial kits were used: porcine sedoheptulose 1,7-bisphosphate (SBP) ELISA kit (YX-190216P); porcine carnosine ELISA kit (M29034); and porcine phosphocreatine ELISA kit (MM-0399O1) (Shanghai Enzyme-linked Biotechnology Co., Ltd. Shanghai, China). Porcine alpha ketoglutaric acid (AKG) ELISA kit (QS47657); porcine hypoxanthine ELISA kit (QS47649) (Qisong Biological, Beijing, China); creatinine assay kit (C011-2-1); glycogen assay kit (A043-1-1); lactic acid content assay kit (A019-2-1); and pyruvic acid assay kit (A081-1-1) (Nanjing Jiancheng Bioengineering Institute, Nanjing, China). All procedures were carried out according to the manufacturer’s instructions.

### 2.6. Metabolite Extraction, Separation, and UHPLC-MS/MS Analysis

Based on meat pH at 45 min, 20 porcine plasma samples with high pH value (HpH; pH = 6.75 ± 0.08, n = 10) and low pH value (HpH; pH = 6.16 ± 0.22, n = 10) for plasma metabolomics analysis. Briefly, 100 μL of plasma samples was placed in the eppendorf tubes and 400 μL of methanol was added to plasma samples, and then vortexed. Tubes were placed in ice-cold water for 5 min and centrifuged at 15,000× *g* at 4 °C for 20 min. The supernatant was transferred to a new tube and LC-MS grade water was added to dilute the methanol to a 53% concentration, followed by centrifugation at 15,000× *g* at 4 °C for 20 min. The supernatant was obtained and injected in the LC-MS/MS system. The UHPLC-MS/MS analysis was completed by Beijing Novogene Co., Ltd. The details of the procedure refer to the previous publication [15].

### 2.7. Data Processing and Metabolite Identification

Peak detection, alignment, and quantitation of raw data were performed in Compound Discoverer 3.1 (Thermo Fisher, Waltham, MA, USA). Peak intensities were normalized to total spectral intensity, and peaks were matched against mzCloud (https://www.mzcloud.org/, accessed on 2 December 2021), mzVault and MassList database to obtain qualitative and quantitative results.

### 2.8. Statistical Analysis

Pearson correlation analysis was performed with GraphPad Prism 8 software. Unpaired Student’s *t*-test was conducted to compare mean values between HpH and LpH sample groups. Principal components analysis (PCA) and partial least squares discriminant analysis (PLS-DA) were performed using metaX [16]. T-test was used to calculate the statistical significance to determine differential metabolites, which were considered when variable importance projection (VIP) value > 1.5 and *p*-value < 0.05 and fold change (FC) ≥ 1.5 or ≤0.66. Volcano plots were used to screen metabolites of interest based on log2 of FC and −log10 of *p*-value using ggplot2 in R language. For heatmap clustering, data were normalized using z-scores of intensity areas of differential metabolites, and plots were generated with the Pheatmap package in R language. Function prediction and pathway enrichment analysis of differential metabolites were conducted based on the KEGG database using MetaboAnalyst (https://www.metaboanalyst.ca/, accessed on 15 June 2022).

## 3. Results

### 3.1. Correlation Analysis among Pork Meat Quality Traits

**Figure 1:**
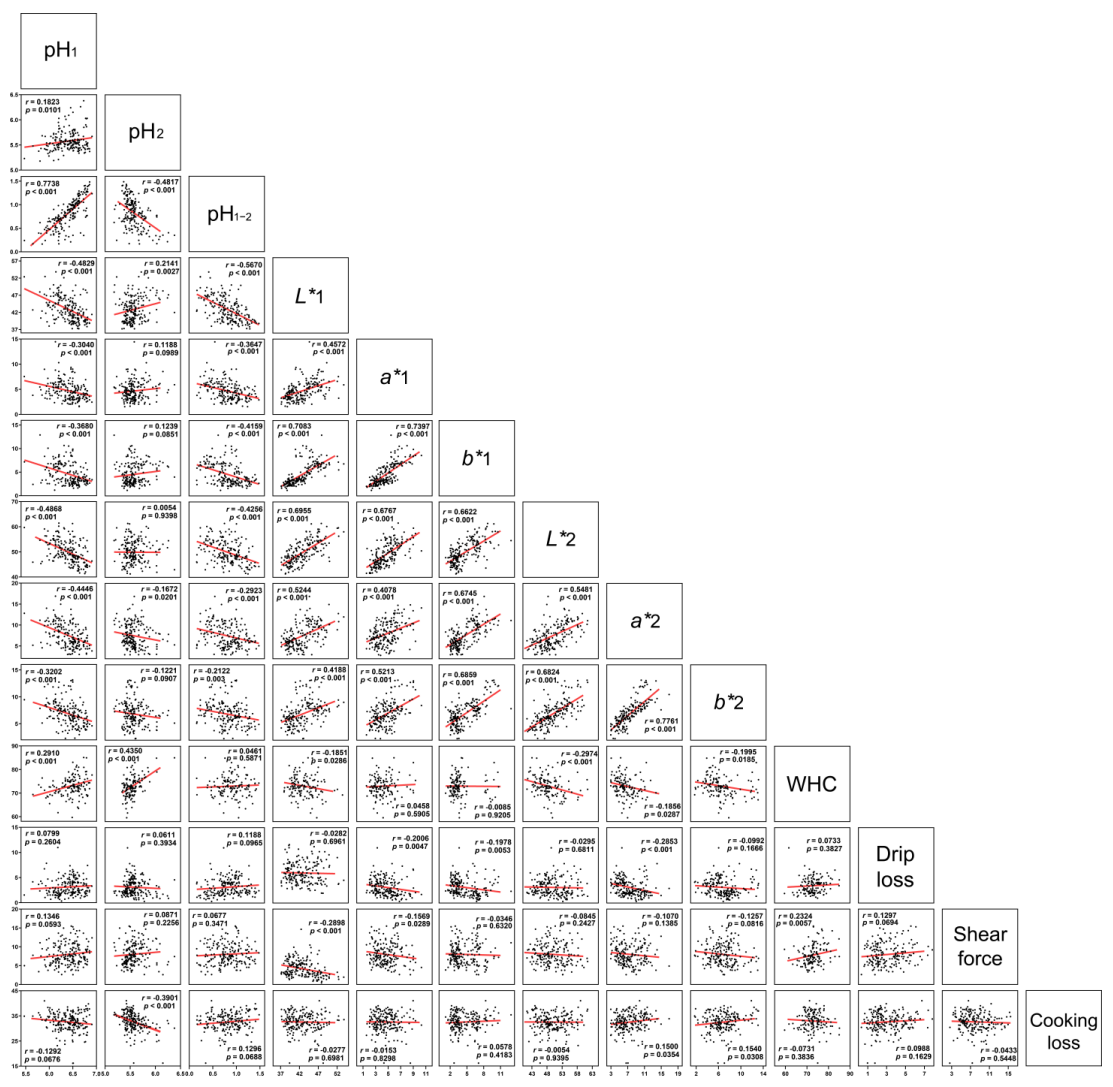
Pearson’s correlation coefficients for pork meat quality traits. pH_1_, L*1, a*1, and b*1 values were measured 45 min postmortem; pH_2_, L*2, a*2, and b*2 values were measured 24 h postmortem; pH_1-2_ corresponded to the difference between pH_1_ and pH_2_ values. WHC: water holding capacity. N = 73–202. Pearson correlation coefficients (*r*) and *p* values were determined by Pearson correlation analysis. Red lines indicate the trend line.

To investigate the influence of pH on pork meat quality, Pearson’s correlation analysis was used on meat quality traits of LTL-based pork meat, and the correlation coefficients are shown in Figure 1. pH_1_ had a strongly positive correlation with pH_1-2_ (*r* = 0.7738; *p* < 0.001) and a significant negative correlation with indicators such as meat color, including L*, a*, and b* values (*r* = −0.4868–−0.3040; *p* < 0.001) at 45 min or 24 h after slaughter. Correlation coefficients between pH_1_ and 2 values and meat color were similar to those between pH_1_ and meat color. Moreover, correlation coefficients between pH_1_ value and drip loss, shear force, cooking loss were not significant (*p* > 0.05). All measured indices of meat color moderately or strongly correlated with one another (0.4078 < *r* < 0.7761). A relatively weak correlation was found between WHC with other quality traits, except pH_2_ (*r* = 0.4350).

### 3.2. Comparison of Pork Meat with Different Quality

Considering that a significant correlation was found between pH_1_ value and other quality traits of pork meat, pH_1_ was used as an indicator to screen among ten plasma samples with high pH (HpH) and ten samples with low pH (LpH). Differences were found in meat quality between HpH and LpH sample groups. All six indicators of meat color were significantly greater in LpH sample group compared with the HpH group (*p* < 0.01) (Table 1). Conversely, drip loss was significantly lower in the HpH group compared with the LpH group (HpH: 2.26 ± 0.60%; LpH: 4.03 ± 1.43%, *p* < 0.01). No significant differences were observed between WHC, shear force, and cooking loss (*p* > 0.05) among both sample groups.

### 3.3. Relationship between Metabolite Profiles in Porcine Plasma and LTL Muscle

**Figure 2:**
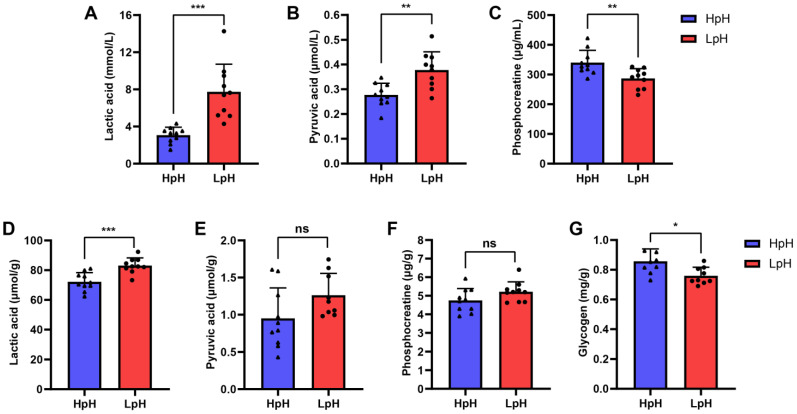
Content of metabolites in porcine plasma and longissimus thoracis et lumborum (LTL) muscle. Lactic acid (**A**), pyruvic acid (**B**), and phosphocreatine (**C**) contents in plasma. Lactic acid (**D**), pyruvic acid (**E**), phosphocreatine (**F**), and glycogen (**G**) contents in LTL muscle. The results are presented as means ± SEM (n = 10). Significant differences were determined by unpaired *t*-test (ns: no significant difference; * *p* < 0.05; ** *p* < 0.01; *** *p* < 0.001). HpH: muscle tissue with high pH; LpH: muscle tissue with low pH.

Subsequently, several metabolites were investigated in porcine plasma and LTL pork meat, including lactic acid, pyruvic acid, and phosphocreatine, which could be reportedly related to pork meat quality [17,18,19]. Besides, we also measured the content of glycogen in LTL muscle. Compared with the HpH group, the contents of lactic acid and pyruvic acid in LpH group were significantly increased in plasma (*p* < 0.01) (Figure 2A,B). However, the content of phosphocreatine in plasma was significantly reduced in the LpH group (*p* < 0.01) (Figure 2C). Interestingly, changing trends in the contents of lactic acid and pyruvic acid in LTL muscle were similar to those found in plasma (Figure 2D). Nevertheless, the contents of pyruvic acid and phosphocreatine in LTL muscle did not statistically change between HpH and LpH sample groups (Figure 2E,F). In addition, the content of glycogen in LTL muscle in HpH group was higher than in LpH group (Figure 2G). Thus, the above results suggested that the contents of certain metabolites in porcine plasma were associated with LTL muscle.

### 3.4. Quality Control of UHPLC-MS/MS Analysis

**Figure 3:**
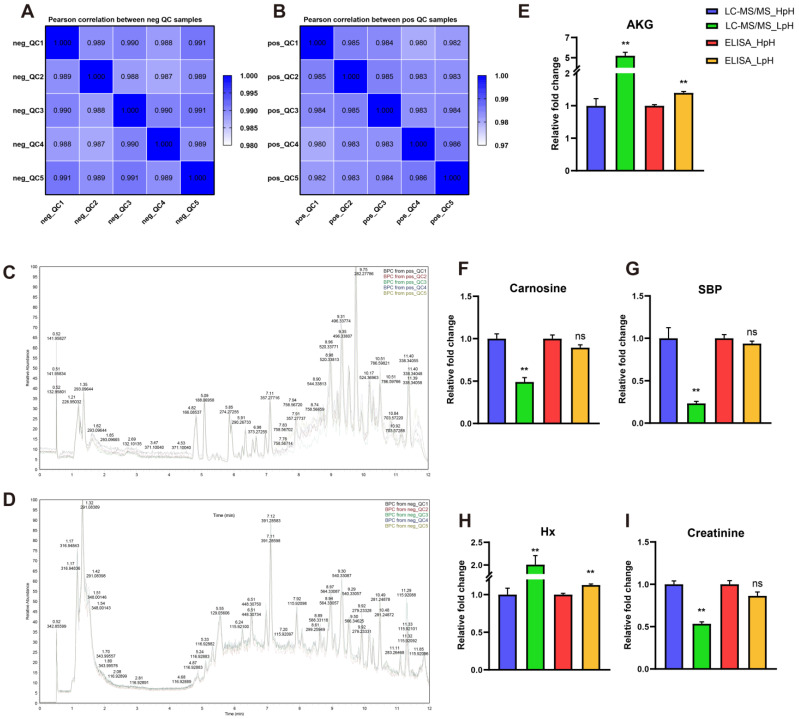
Quality control (QC) of metabolomics analysis based on ultra-high-performance liquid chromatography coupled with mass spectrometry (UHPLC-MS/MS). Heatmap of correlation between base peak intensity chromatograms of QC samples in positive (**A**) or negative (**B**) ion modes. Base peak chromatogram (BPC) of QC samples in positive (**C**) or negative (**D**) ion modes. Relative fold changes of several metabolites in the plasma of porcine muscle with high pH (HpH) and low pH (LpH) using ultra-high-performance liquid chromatography coupled with mass spectrometry (UHPLC-MS/MS) or immunoassay (ELISA). (**E**) Alpha ketoglutaric acid (AKG); (**F**) carnosine; (**G**) sedoheptulose 1,7-bisphosphate (SBP); (**H**) hypoxanthine (Hx); (**I**) creatinine. The results are presented as means ± SEM (n = 10). Significant differences were determined by unpaired *t*-test (ns: no significant difference, ** *p* < 0.01).

To further explore differences in the metabolite profile in porcine plasma in HpH and LpH samples, an untargeted metabolomic approach based on UHPLC-MS/MS was used. In order to evaluate UHPLC-MS/MS data quality, all plasma samples were pooled using an equal volume to prepare five quality control (QC) samples. A heatmap analysis of correlation coefficients and base peak chromatograms of QC samples in positive (Figure 3A,C) and negative (Figure 3B,D) ion modes revealed that QC samples had good repeatability, thus indicating the reliability and stability of obtained metabolomic data.

After further analysis of raw data, a total of 899 metabolites were identified in all samples, among which 531 metabolites were identified in positive-ion mode and 368 in negative-ion mode (Table S1). Moreover, the content of several metabolites in plasma was verified by ELISA to confirm metabolomic data. A significant increase in the contents of alpha ketoglutaric acid (AKG) and hypoxanthine (Hx) was found in the LpH group, as demonstrated by ELISA and UHPLC-MS/MS (Figure 3E,H). The contents of carnosine, sedoheptulose 1,7-bisphosphate (SBP), and creatinine in LpH were significantly lower compared with those in HpH, as determined by UHPLS-MS/MS. In contrast, no significant differences were observed in the contents of carnosine, SBP, and creatinine when determined by ELISA, but they showed similar decreasing trends as demonstrated by UHPLC-MS/MS (Figure 3F,G,I).

### 3.5. Multivariate Statistical Analysis

**Figure 4:**
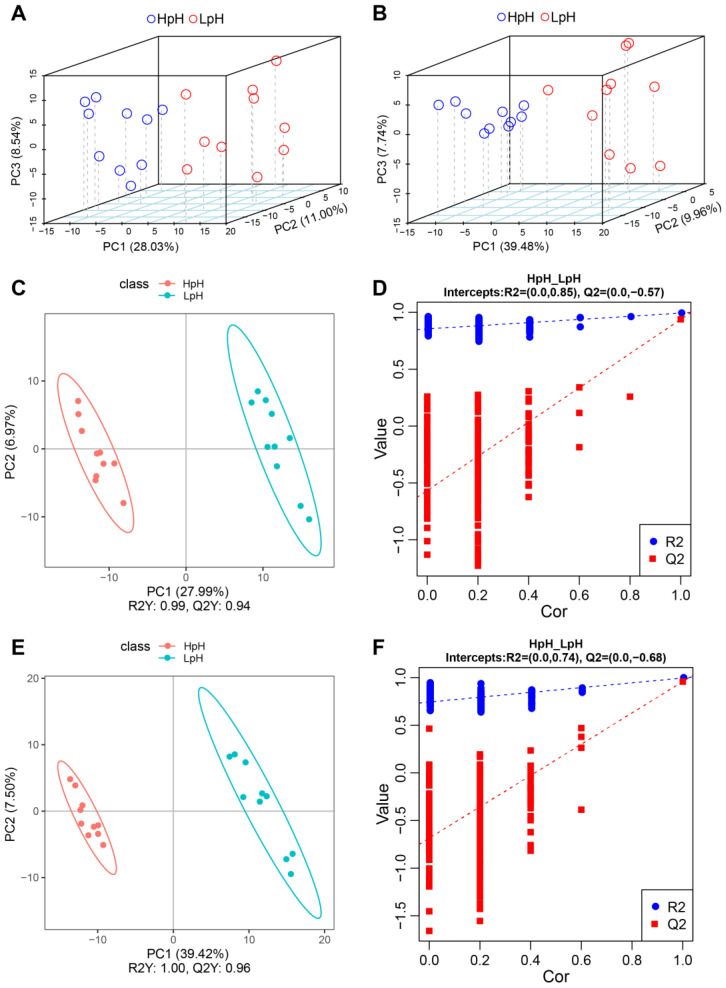
Three-dimensional score plots of principal component analysis (PCA) and partial least-squares discriminant analysis (PLS-DA) of metabolomic data in the plasma of porcine muscle with high pH (HpH) and low pH (LpH) in positive (**A**) and negative (**B**) modes. Score plot of PCA in positive (**C**) and negative (**E**) modes. Validation of the PLS-DA model using permutation tests in positive (**D**) and negative (**F**) modes.

Multivariate statistical analysis is an effective strategy to reduce the complexity of multidimensional data matrices. In the present study, an unsupervised PCA model was constructed to determine the separation of metabolite profiles in porcine plasma in different sample groups. As shown in the PCA plot (Figure 4), HpH samples clustered separately from LpH samples when analyzed either in positive (Figure 4A) or negative (Figure 4B) ion modes.

To further differentiate the two sample groups, a supervised partial least-squares discriminant analysis (PLS-DA) was conducted on metabolomic data. PLS-DA evaluation parameters R2 and Q2 were obtained by cross-validation to determine the reliability of the PLS-DA model, which were as follows: R2 = 0.99 and Q2 = 0.94 in positive mode (Figure 4C), and R2 = 1 and Q2 = 0.96 in negative mode (Figure 4E). In permutation tests, R2 and Q2 values either in positive (Figure 4D) or negative (Figure 4F) modes were lower than the values obtained in the PLS-DA model.

### 3.6. Screening and Identification of Differential Metabolites

Subsequently, differential metabolites were identified based on FC, *p*, and variable importance projection (VIP) values, which were calculated using the PLS-DA model. Screening criteria were VIP and FC values > 1.5 or FC value < 0.666 and *p* value < 0.05.

**Figure 5:**
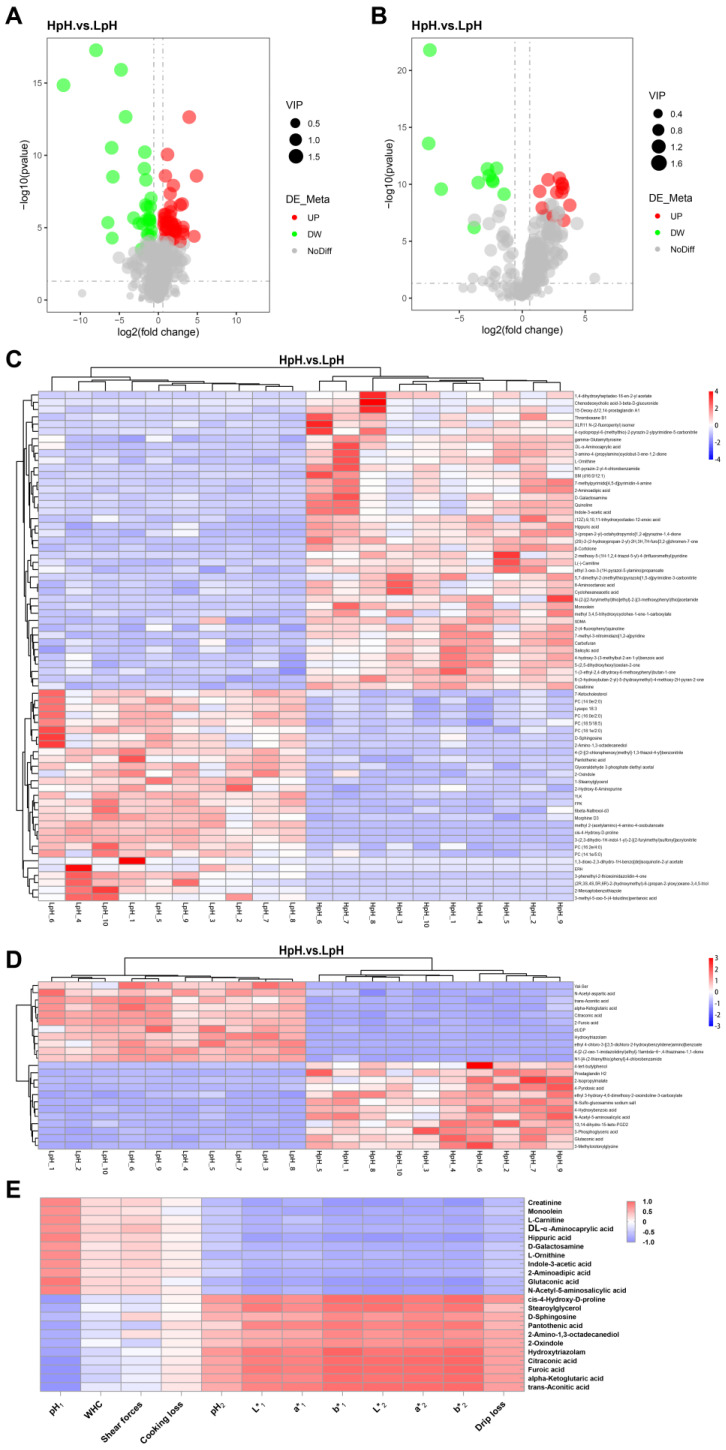
Volcano plot and hierarchical cluster heatmap of differential metabolites in porcine plasma. (**A**) Volcano plot of differential metabolites identified in positive-ion mode. (**B**) volcano plot of differential metabolites identified in negative-ion mode. Each dot represents a single metabolite; red dots indicate upregulated metabolites; blue dots indicate downregulated metabolites. (**C**) Hierarchical cluster heatmap of differential metabolites identified in positive-ion mode. (**D**) Hierarchical cluster heatmap of differential metabolites identified in negative-ion mode. (**E**) Heatmap based on Pearson correlation coefficients between several differential metabolites and meat quality traits.

In total, 93 differential metabolites were identified, among which 70 metabolites were identified in positive-ion mode and 23 in negative-ion mode (Table S2). In addition, a volcano plot was generated to enable the visualization of differential metabolites between the two sample groups; in HpH, 41 metabolites were upregulated with 26 downregulated in positive-ion mode (Figure 5A); in negative-ion mode, 12 were upregulated and 11 were downregulated (Figure 5B).

Furthermore, a hierarchical clustering heatmap was constructed to enable the visualization of changes in differential metabolites between sample groups. As depicted in Figure 5C,D, samples in the same group clustered together, indicating that differential metabolites enabled strong sample discrimination. Figure 5E depicts the correlation between several differential metabolites and meat quality.

### 3.7. KEGG Enrichment Analysis of Differential Metabolites

**Figure 6:**
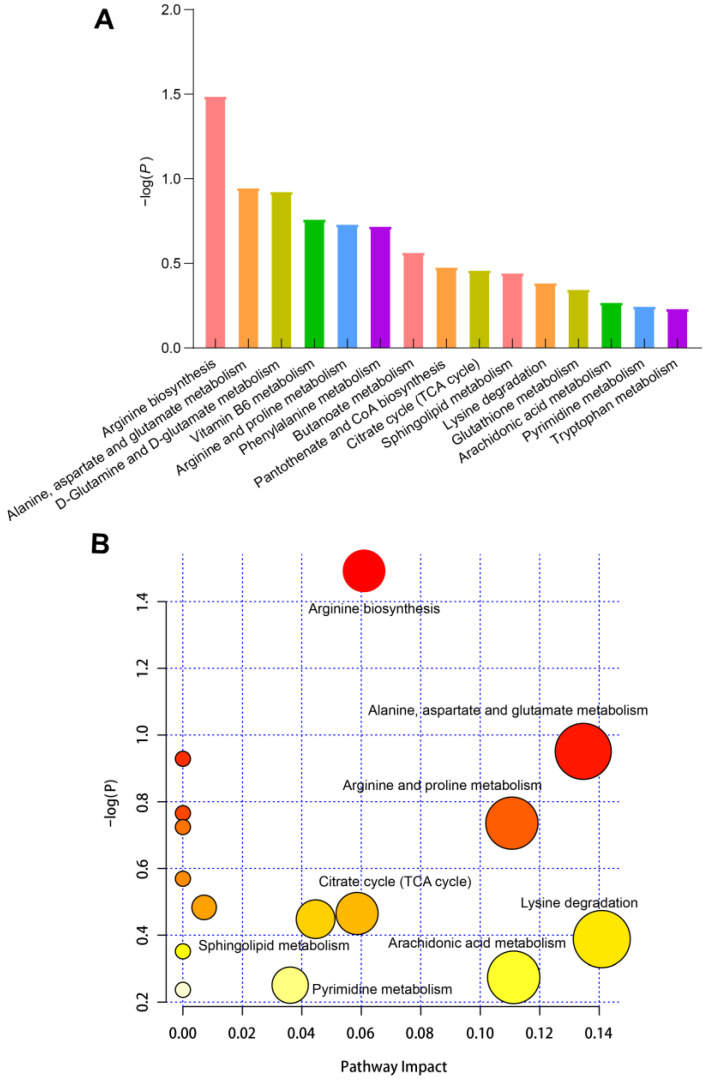
Kyoto Encyclopedia of Genes and Genomes (KEGG) enrichment analysis of differential metabolites in the plasma. (**A**) Bar graphs of the top fifteen most enriched metabolic pathways. (**B**) Bubble plot of top most enriched pathways. Dot size and color shade are positively related to the impact on the respective metabolic pathway.

Finally, KEGG enrichment analysis was conducted to identify metabolic pathways in which differential metabolites are involved. A total of 15 metabolic pathways were identified, i.e., arginine biosynthesis; alanine, aspartate, and glutamate metabolism; D-glutamine and D-glutamate metabolism; vitamin B6 metabolism; arginine and proline metabolism; phenylalanine metabolism; butanoate metabolism; pantothenate and CoA biosynthesis; tricarboxylic acid (TCA) cycle; sphingolipid metabolism; lysine degradation; glutathione metabolism; arachidonic acid metabolism; pyrimidine metabolism; and tryptophan metabolism (Figure 6). Thus, the identified enriched pathways were associated mainly with amino acid metabolism and energy substance metabolism.

## 4. Discussion

Meat quality parameters (e.g., pH, meat color, drip loss, and shear force) are important in the evaluation of pork meat quality. In particular, color is a critical indicator of consumer acceptance related to meat quality, freshness, and safety [20]. In the present study, a correlation analysis was conducted among pork meat quality traits, which revealed that pH of pork meat 45 min postmortem strongly correlated with other meat quality traits. Thus, pH at 45 min after slaughter was the most important indicator reflecting pork meat quality. Pyruvate produced in muscle tissue during glycolysis is preferentially metabolized into lactate by lactate dehydrogenase after slaughter, which results in pH decline in muscle tissue. The influence of muscle tissue pH on meat quality parameters has been extensively reported, and is known to impact meat flavor, freshness, tenderness, shelf-life, and color [21,22]. A previous study reported that muscle pH was negatively correlated with L* value [23], which agreed with the findings of the present study. Previous studies have reported that low pH values in muscle tissue might affect myoglobin oxygenation. In this context, a layer of oxymyoglobin develops on the surface of meat, which increases the brightness and color saturation of meat [24]. Welzenbach et al. reported that a high rate of glycolysis results in a high meat color L* value [25]. Furthermore, taken together, these observations may explain the negative correlation between pork meat pH and meat color values. Additionally, in the present study, pH_1_ and pH_2_ values significantly correlated with WHC of pork meat. Conversely, drip loss, shear force, and cooking loss did not significantly associate with other pork meat quality traits; this may be attributed to the relatively small sample size used in the current study. Overall, the results discussed herein support previous findings reported in the literature.

Generally, after slaughter, cellular metabolism in muscle tissue shifts from aerobic to anaerobic catabolism of glycogen [26]. This shift causes pH of pork meat to subsequently decline as a result of lactate production and H^+^ accumulation. The decline rate of muscle pH after slaughter and final pH of meat greatly affects meat quality [27]. When meat pH decreases excessively, the denaturation of sarcoplasmic and myofibrillar proteins is induced, further leading to a paler color and low WHC; in severe cases, pale, soft, and exudative (PSE) meat is generated [28]. In the present study, a total of 20 samples with high or low pH (HpH and LpH, respectively) were compared in terms of the difference in meat quality. Meat color indexes of HpH samples were significantly lower than those of LpH (*p* < 0.01), which strengthens the negative correlation observed between pork meat pH and color. Meat pH value is known to be negatively related to drip loss; however, a significant correlation was not observed between these traits in the present study based on Pearson correlation analysis. Interestingly, drip loss in HpH group decreased significantly (*p* < 0.01). In fact, the magnitude of correlation between drip loss and pH differed widely among studies [29]. It is known that excessive drip loss in pork meat implies not only economic but also nutrient losses. Therefore, an extremely low pH value in pork meat could be detrimental to drip loss, which in turn reduces meat quality.

Subsequently, the content of several differential metabolites in porcine plasma and LTL muscle tissue in HpH and LpH samples were evaluated. A higher content of lactic acid was found in LpH compared with that in HpH in both the plasma and LTL muscle. No difference was found in the contents of pyruvic acid and phosphocreatine in LTL muscle between the two sample groups. However, the glycogen content was lower in LpH. As anaerobic glycolysis is the major metabolic pathway in muscle tissue after slaughter, glycogen is initially converted into pyruvic acid, then further converted into lactic acid under anaerobic conditions, which might explain the higher lactic acid content and lower glycogen content in LpH. Although no significant differences were found in pyruvic acid content in LTL muscle, an upward trend was found for pyruvic acid content in LTL muscle of LpH samples. Moreover, the content of phosphocreatine did not significantly change in LTL muscle. Recent studies have reported that phosphocreatine hydrolysis led to a pH increase in beef longissimus lumborum, although its influence was small [19]. Thus, the above results suggest that the contents of metabolites in porcine plasma reflect those in LTL muscle to some extent. Therefore, untargeted plasma metabolomics based on UHPLC-MS/MS was conducted to investigate differences between sample groups.

In the present study, 531 and 368 metabolites were detected in samples in positive- and negative-ion modes, respectively. The inclusion of QC samples and building correction models based on QC samples is a commonly used methodology in metabolomic studies [30]. Herein, a good overlap was found between base peak chromatogram and QC samples. Moreover, the stability of the analysis was high, and the quality of metabolomic data was acceptable for downstream analyses.

Herein, the content of several metabolites in porcine plasma was determined by ELISA to verify UHPLC-MS/MS metabolomic data. The relative contents of AKG and Hx were increased in LpH as demonstrated either by UHPLC-MS/MS or ELISA. In addition, the contents of carnosine, SBP, and creatinine were determined by ELISA, which followed a similar changing trend as demonstrated by UHPLC-MS/MS, which overall indicated that metabolomic data were accurately determined.

Moreover, PCA revealed clear differences between HpH and LpH groups. Subsequently, PLS-DA was applied to identify metabolites responsible for the differences observed in samples as revealed by PCA. With significance threshold based on *p* value < 0.05, and differential thresholds as FC value > 1.5 or < 0.666 and VIP value > 1.5. In total, 70 and 23 differential metabolites were found in positive- and negative-ion modes, respectively; among these, 41 were upregulated and 29 were downregulated in positive-ion mode, whereas 12 were upregulated and 11 were downregulated in negative-ion mode. Furthermore, Pearson correlation analysis revealed that several metabolites were strongly correlated with pork meat quality traits, such as L-carnitine, creatinine, sphingosine, L-ornithine, and D-galactosamine, among others. In a previous study, the serum metabolome in Shaziling and Yorkshire pigs was investigated, revealing that L-carnitine was negatively correlated with lean percentage and L* and b* values [31], which agreed with the results of the present study. L-carnitine is important for fatty acid oxidation in mitochondria [32]. Moreover, sphingosine was significantly correlated with pork meat quality in the present study. Previous studies have identified sphingosine as a marker for predicting chicken meat quality [33].

Moreover, KEGG pathway enrichment analysis was applied to differential metabolites identified in pork meat. Differential metabolites were mainly involved in pathways related to amino acid metabolism (including arginine biosynthesis; alanine, aspartate, and glutamate metabolism; arginine and proline metabolism; and phenylalanine metabolism) as well as energy metabolism (including TCA cycle and fatty acid metabolism). These findings suggest that changes in energy metabolism and amino acid metabolism may lead to differences in pork meat quality. Importantly, only a correlation analysis was conducted between metabolites and meat quality traits in the present study, but these may not be directly responsible for the changes in meat quality.

## 5. Conclusions

Collectively, the findings of the present study enlarge the current understanding on an existing correlation among pork meat quality traits. In particular, meat pH value was identified as the most important quality trait for pork meat. In addition, metabolites in the plasma of porcine LTL muscle with high or low pH values were identified by untargeted metabolomics using UHPLC–MS/MS, and the identified differential metabolites could potentially be used as biomarkers for predicting meat quality. However, further studies on a larger sample size are necessary to confirm the findings discussed herein. Taken together, the present study provided fundamental data for exploring plasma biomarkers to predict pork meat quality.

## Figures and Tables

**Table 1:** Quality traits of porcine longissimus thoracis et lumborum (LTL) muscle with different pH values.

Meat Quality Trait	High pH	Low pH	*p* Value
Body weight	128.5 ± 14.74	138 ± 15.04	ns
pH_1_	6.75 ± 0.08	6.16 ± 0.22	**
pH_2_	5.40 ± 0.05	5.51 ± 0.07	*
L*1	39.07 ± 0.98	44.40 ± 2.10	*
a*1	3.96 ± 0.90	6.89 ± 1.29	**
b*1	3.11 ± 0.62	9.25 ± 0.91	**
L*2	43.07 ± 2.54	56.57 ± 2.70	**
a*2	5.85 ± 1.00	10.27 ± 0.99	**
b*2	4.40 ± 1.08	11.95 ± 0.47	**
WHC (%)	73.57 ± 1.99	71.64 ± 3.53	ns
Drip loss (%)	2.26 ± 0.60	4.03 ± 1.43	*
Shear forces (N)	7.16 ± 1.26	6.39 ± 2.09	ns
Cooking loss (%)	35.40 ± 3.90	36.04 ± 2.37	ns

Note: pH_1_, L*1, a*1, and b*1 were measured at 45 min postmortem; pH_2_, L*2, a*2, and b*2 were measured at 24 h postmortem; significance was based on two-tailed test. WHC: water holding capacity; the results are expressed as means ± SEM, ns: no significant difference; N = 10 per group. * *p* < 0.01; ** *p* < 0.001.

## Data Availability

For the remaining data that may be relevant, the corresponding authors can be contacted.

## Supplementary Materials

The following supporting information can be downloaded at https://www.mdpi.com/article/10.3390/foods11244005/s1. Table S1: The list of all annotated metabolites; Table S2: Number of differential metabolites between pork meat samples with HpH and LpH.
