# Supplementary material for: Plasma Metabolomic Profiling Reveals Preliminary Biomarkers of Pork Quality Based on pH Value
Source: Foods. 2022 Dec 11;11(24):4005. doi: 10.3390/foods11244005 (PMC9778167; doi:10.3390/foods11244005)
Supplement: Supplementary file 1 [file foods-11-04005-s001.zip › Table S2.pdf]

Table S2. Number of differential metabolites between pork meat samples with HpH and LpH.

Ion mode	Total	Upregulated	Downregulated
Positive	531	41	29
Negative	368	12	11
